# Prognostic role of elevated VEGF in sepsis: A systematic review and meta-analysis

**DOI:** 10.3389/fphys.2022.941257

**Published:** 2022-07-22

**Authors:** A-ling Tang, Yu Peng, Mei-jia Shen, Xiao-yu Liu, Shan Li, Meng-chen Xiong, Nan Gao, Tian-peng Hu, Guo-qiang Zhang

**Affiliations:** ^1^ Graduate School, Beijing University of Chinese Medicine, Beijing, China; ^2^ Department of Emergency, China-Japan Friendship Hospital, Beijing, China; ^3^ Graduate School of Peking Union Medical College, Beijing, China

**Keywords:** sepsis, VEGF, meta-analysis, systematic review, prognostic

## Abstract

**Background:** The incidence and mortality of sepsis are increasing year by year, and there is still a lack of specific biomarkers to predict its prognosis. Prognostic value of vascular endothelial growth factor (VEGF) in predicting the severity and mortality of sepsis has been gradually discovered.

**Methods:** Literature was searched through Embase, PubMed, Web of Science, China National Knowledge Infrastructure(CNKI) and Cochrane Library databases in March 2022. Observational studies, evaluating the impact of VEGF in sepsis outcomes (mortality and severity) are included in this meta-analysis. Risk of bias was assessed with the Newcastle-Ottawa Scale (NOS). Sensitivity and publication bias analyses were also assessed. Meta-regression analysis were performed to identify the potential sources of heterogeneity.

**Result:** A total of 1,574 articles were retrieved from the systematic literature search. We included 20 studies for qualitative and quantitative analysis. Deceased and critically ill patients had higher baseline VEGF levels than survivors and non-severe patients. The pooled sensitivity and specificity for VEGF predicts sepsis mortality were 0.79and 0.76, respectively. the area under the SROC curve was 0.83.

**Conclusion:** High VEGF are associated with poor clinical outcomes for patients diagnosed with sepsis. This study was recorded on PROSPERO, under the registration ID: CRD42022323079.

## 1 Introduction

Sepsis is life-threatening organ dysfunction caused by a dysregulated host response to infection (Singer et al., 2016), which has the clinical characteristics of complex pathogenesis, rapid disease development and high mortality. Currently, there are no robust biomarkers that can effectively predict the prognosis of sepsis. There are no robust biomarkers that can stratify patients to the risk of sepsis complications (Alves et al., 2011), which brings great challenges to clinical work. In patients with sepsis, damage to microvascular endothelial cells can not only cause vascular leakage and edema but can also cause serious complications such as shock, microthrombosis, and multiple organ failure (MOF) (Lammers et al., 2008). Biomarkers related to sepsis prognosis include inflammation related (CRP, IL-6, IL-8, IL-18, PCT), organ dysfunction related (Lactate), vascular endothelial injury related (VEGF, Ang2/ANG-1, SFLT-1), and Oxidative Damage related (MDA, F2-ISOprostanes), etc (Vera et al., 2015). Endothelial dysfunction is associated with the pathogenesis and progression of systemic inflammatory responses (SIRS) (Shapiro et al., 2010). Several biomarkers related to vascular endothelial function have attracted the attention of researchers, such as Angiotensin 1 (Ang 1), Angiotensin 2 (Ang 2), VEGF, Soluble FMS-like tyrosine kinase 1(SFLT-1) (Pregernig et al., 2019)^,^ (Shapiro et al., 2020)^,^ (Russa et al., 2019). VEGF is an endothelial growth factor, which is involved in angiogenesis in various physiological and pathological states. Increased vascular permeability is an important pathophysiological mechanism of sepsis, and VEGF has a strong role in promoting vascular permeability (Senger et al., 1983). Compared with other biomarkers, it may better reflect the progression of sepsis from the pathophysiological mechanism. Blockade of VEGF-A reduces mortality in mice with sepsis, making it a potential target for the treatment of vascular barrier disruption in sepsis (Smadja et al., 2012). There was also a clinical study that found VEGF could distinguish between severe sepsis and non-infectious organ failure (Hauschildt et al., 2020). In several clinical studies, the impact of VEGF on the severity and mortality of sepsis patients has been confirmed. Therefore, this systematic review and meta-analysis evaluated the prognostic value of elevated VEGF in sepsis.

## 2 Materials and methods methods

This review followed the Preferred Reporting Items for Systematic Reviews and Meta-Analyses (PRISMA) Statement (Page et al., 2021).

### 2.1 Search strategy

Literature was searched through Embase, PubMed, Web of Science, China National Knowledge Infrastructure (CNKI) and Cochrane Library databases in March 2022, and no language or date restrictions were applied. Complete a systematic search on a combination of title, abstract and Medical Subject Headings (MeSH). The full search strategy is detailed in the Supplementary Table S1.

### 2.2 Study selection

**FIGURE 1:**
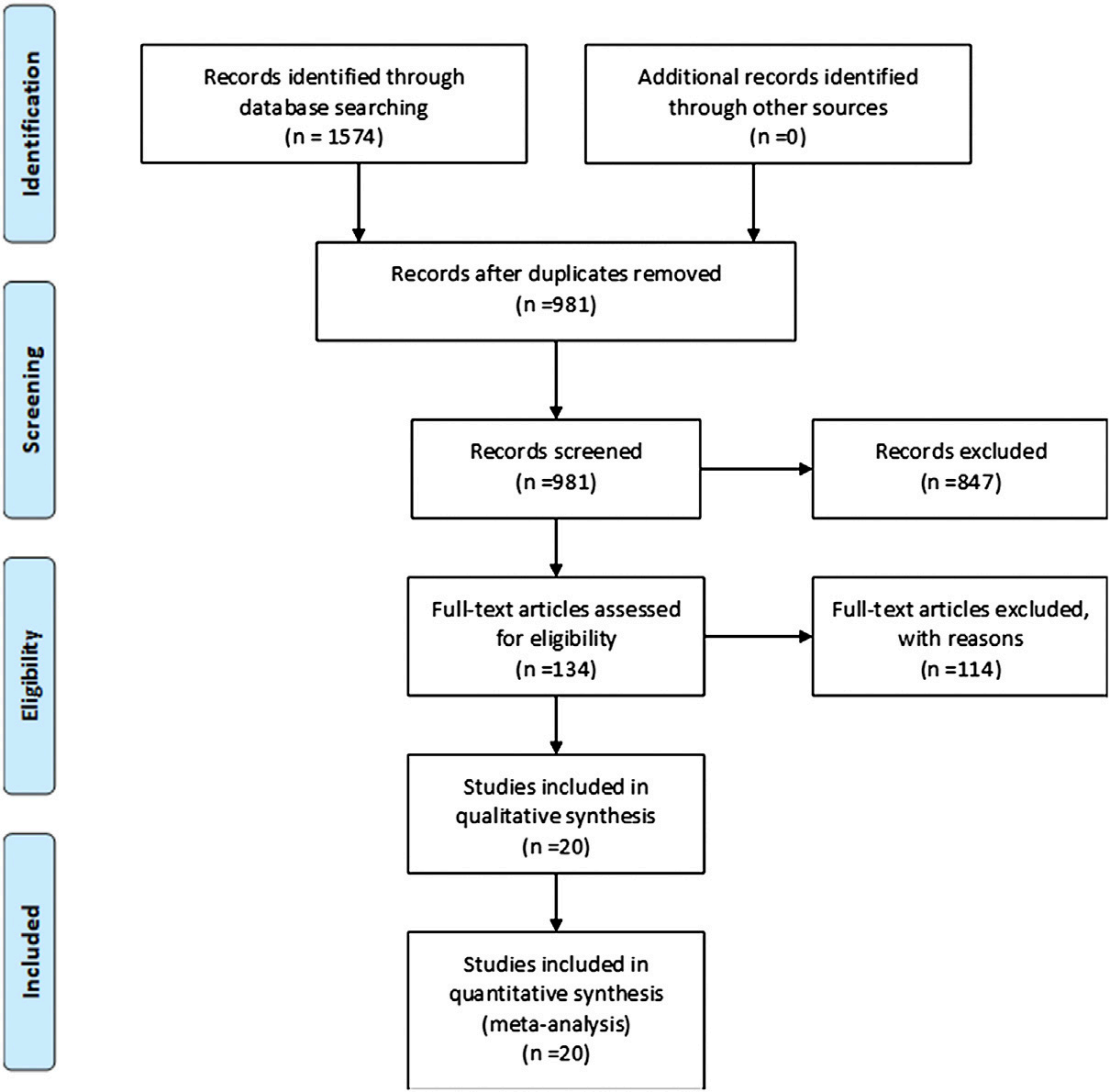
PRISMA flow diagram of study selection.

Two authors independently selected studies. In case of disagreement, it was firstly resolved by discussion between the two authors. If there is still disagreement, a third author is consulted. The details of selection of articles in accordance with the PRISMA guidelines are shown in Figure 1.

### 2.3 Inclusion and exclusion criteria

The following inclusion criteria were used:1) Human subjects; 2) Clinical studies; 3) observational studies; 4) Research has a clear definition of sepsis (sepsis1-3) (Singer et al., 2016); 5) Prognostic information was associated with all-cause mortality and disease severity in patients with sepsis; The exclusion criteria were, as follows: 1) *In vitro* experiments, animal and interventional experiments; 2) Reviews, commentaries, letters, case reports, correspondences, conference abstracts, expert opinions; 3) Duplicate articles;

### 2.4 Assessment of risk of bias

The internal quality of included studies was assessed using the NOS (Wells et al., 2014) by two independent reviewers. NOS includes the following components: selection of study groups; comparability of groups; evaluation of exposures or outcomes. These three parts include 4, 2, and three sub-aspects. For each item, if the answer is “yes”, you can give the study a star. According to the score, studies are divided into three qualities: high (7–9), medium (4–6) and low (0–3).

### 2.5 Data extraction and analysis

Information retrieved from all studies involved: 1) general study information: author, year, country, study design; 2) patient characteristics: sample size; 3) VEGF measurement: time point of measurement, assay method; 4) severity of sepsis: sepsis, septic shock, severe sepsis, sepsis with MOF; 5) mortality: follow-up duration; 6) outcome measures: VEGF concentration in survivors and nonsurvivors, VEGF concentration in sepsis and severe sepsis (septic shock, severe sepsis, sepsis with MOF), the area under the receiver operating characteristic (ROC) curve was used to calculate the sensitivity and specificity of VEGF in predicting mortality.Where possible, data is retrieved directly from publications. We will obtain the data indirectly through the pictures or computational data set provided by the author, if the data cannot be obtained directly (Wan et al., 2014). Data were independently extracted by two authors using a data extraction form containing patient characteristics and outcome data.

Data synthesis was performed using RevMan software 5.4 and Stata 12. For continuous variables, standardized mean difference (SMD) and 95% CI between two groups were calculated. To test heterogeneity, I^2^ statistics was computed, and a χ^2^ test was performed. When there is significant heterogeneity (I^2^ ≥ 50%), random effect model is used. Meta-regression analysis were performed to identify the potential sources of heterogeneity. When the heterogeneity was low (I^2^<50%), the fixed effect model was used. Sensitivity analyses were performed on all results to test the stability of the meta-analysis results. Funnel plot symmetry and Egger test was used to assess the risk of publication bias for each study. Significance level for all two-sided *p* values was set at less than 0.05.

## 3 Results

### 3.1 Study selection and characteristics

A total of 1,574 articles were retrieved from the systematic literature search. We included 20 studies for qualitative and quantitative analysis., with a total of 2,242 participants. (Figure 1). A total of 16 prospective studies and four retrospective studies were included. All of the studies completed VEGF concentration testing within 3 days of admission or enrollment in the study. We summarize the general information of the study in Table 1: author, year, country, study design, sample size, assay used, mortality follow-up, and NOS scores. Thirteen high quality studies (Liu et al., 2009; Davis et al., 2010; Yang et al., 2011; Xu et al., 2013; Lin et al., 2015; Amalakuhan et al., 2016; Hou et al., 2017; Patry et al., 2018a; Patry et al., 2018b; Almasy et al., 2020; Li, 2020; Seol et al., 2020; Whitney et al., 2020) and seven medium quality studies (Karlsson et al., 2008; Zhang et al., 2012; Jiang et al., 2014; Tian et al., 2015; Akabawy et al., 2016; Koskela et al., 2017; Xu, 2021) were included, and the scoring details of NOS are shown in Table 2.

**TABLE 1:** Characteristics of all included studies.

Author	Year	Country	Study design	N	Assay	Outcome	NOS Score
Xu, (2021)	2021	China	Retrospective	68	Not mention	28-day mortality	6
Almasy et al., 2020	2020	Romania	Prospective	107	ELISA (R&D Systems)	Septic shock	8
Li, (2020)	2020	China	Retrospective	52	ELISA (R&D Systems)	30-days mortality	8
Whitney et al., 2020	2020	America	Prospective	166	Multiplex immunoassay (Meso Scale Discovery)	Septic shock	7
Seol et al., 2020	2020	South Korea	Retrospective	145	Multiplex immunoassay (Meso Scale Discovery)	28-day mortality	8
Patry et al., 2018a	2018	Germany	Prospective	30	ELISA (R&D Systems)	28-days mortality	8
Patry et al., 2018b	2018	Germany	Prospective	30	ELISA (R&D Systems)	28-day mortality	8
Hou et al., 2017	2017	America	Prospective	605	ELISA (R&D Systems)	60-day mortality	7
Koskela et al., 2017	2017	Finland	Prospective	44	Multiplex immunoassay (Millipore Corporation)	30-day mortality	6
Amalakuhan et al., 2016	2016	America	Prospective	48	Multiplex immunoassay (Luminex)	MOF	8
Akabawy et al., 2016	2016	Egypt	Prospective	64	ELISA	ICU mortality	5
Lin et al., 2015	2015	China	Prospective	96	ELISA (Sekisui Diagnostics)	Hospital mortality	7
Tian et al., 2015	2015	China	Prospective	32	ELISA (Pharma)	Hospital mortality	6
Jiang et al., 2014	2014	China	Prospective	135	ELISA (R&D Systems)	28-day mortality	6
Xu et al., 2013	2013	China	Prospective	123	ELISA (R&D Systems)	28-day mortality	8
Zhang et al., 2012	2012	China	Prospective	59	ELISA	28-day mortality	6
Yang et al., 2011	2011	China	Prospective	81	ELISA (R&D Systems)	28-day mortality	8
Davis et al., 2010	2010	Australia	Prospective	83	ELISA (R&D Systems)	Severe sepsis	8
Liu et al., 2009	2009	China	Retrospective	29	ELISA (ADL)	28-day mortality	8
Karlsson et al., 2008	2008	Finland	Prospective	245	ELISA (R&D Systems)	ICU mortality	6

**TABLE 2:** NOS scores.

Study	Selection				Comparability of Cases and Controls on the Basis of the Design or Analysis	Exposure			Scores
	Adequate definition of cases	Representativeness of the cases	Selection of controls	Definition of controls		Ascertainment of exposure	Same method of ascertainment for cases and controls	Non-response rate	
Xu, (2021)	★☆	★	-	★	-	★	★	★	6
Almasy et al., 2020	★	★	-	★	★★	★	★	★	8
Li, (2020)	★	★	-	★	★★	★	★	★	8
Whitney et al., 2020	★	★	-	★	★★	★	★	-	7
Seol et al., 2020	★	★	-	★	★★	★	★	★	8
Patry et al., 2018a	★	★	-	★	★★	★	★	★	8
Patry et al., 2018b	★	★	-	★	-	★	★	★	6
Hou et al., 2017	★	★	-	★	★★	★	★	★	7
Koskela et al., 2017	★	★	-	★	-	★	★	★	6
Amalakuhan et al., 2016	★	★	-	★	★★	★	★	★	8
Akabawy et al., 2016	★	★	-	★	--	★	★	-	5
Lin et al., 2015	★	★	-	★	★★	★	★	★	7
Tian et al., 2015	★	★	-	★		★	★	★	6
Jiang et al., 2014	★	★	-	★	-	★	★	★	6
Xu et al., 2013	★	★	-	★	★★	★	★	★	8
Zhang et al., 2012	★	★	-	★	-	★	★	★	6
Yang et al., 2011	★	★	-	★	★★	★	★	★	8
Davis et al., 2010	★	★	-	★	-	★	★	★	6
Liu et al., 2009	★	★	-	★	-	★	★	★	6
Karlsson et al., 2008	★	★	-	★	★★	★	★	★	8

### 3.2 VEGF and sepsis mortality

**FIGURE 2:**
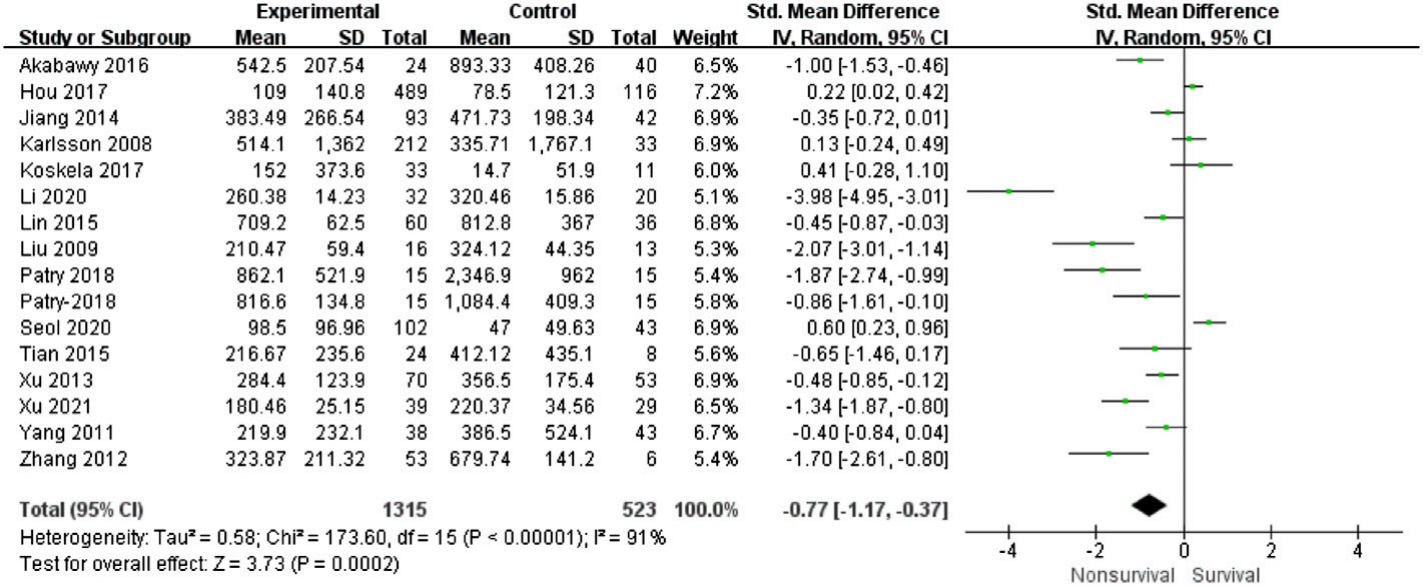
Forest plots of VEGF (nonsurvivors-survivors).

16 studies (Karlsson et al., 2008; Liu et al., 2009; Yang et al., 2011; Zhang et al., 2012; Xu et al., 2013; Jiang et al., 2014; Lin et al., 2015; Tian et al., 2015; Akabawy et al., 2016; Hou et al., 2017; Koskela et al., 2017; Patry et al., 2018a; Patry et al., 2018b; Li, 2020; Seol et al., 2020; Xu, 2021) reported baseline VEGF concentrations in sepsis survivors and non-survivor. Most studies confirmed that VEGF was significantly higher in the nonsurvivable group than in the survivable group. However, a number of studies have shown the opposite. Due to the high heterogeneity between studies (I^2^ = 91%, *p* < 0.00001), we used the random effects model. Meta-analysis showed significant differences in VEGF between the survival and non-survival groups (SMD = -0.77, 95%CI-1.17∼-0.37, *p* = 0.0002). (Figure 2). This suggests that high VEGF is associated with a high risk of mortality. Meta-regression analyses based on the confounding factors such as research starting year, nations, follow-up time of mortality, sample size, sampling time, severity of sepsis, and NOS scores were conducted. The results showed that sample size (>100 vs ≤ 100) may be the source of heterogeneity (*p* = 0.03). Other variables had no significant correlation with VEGF. (Supplementary Figure S1). Sensitivity analysis showed that the results remained stable after sequentially removing a single study. In addition, the funnel plot is asymmetric (Supplementary Figure S2), Egger test results show that *p* = 0.001, indicating that there may be publication bias between studies.

### 3.3 VEGF and severity of sepsis

**FIGURE 3:**
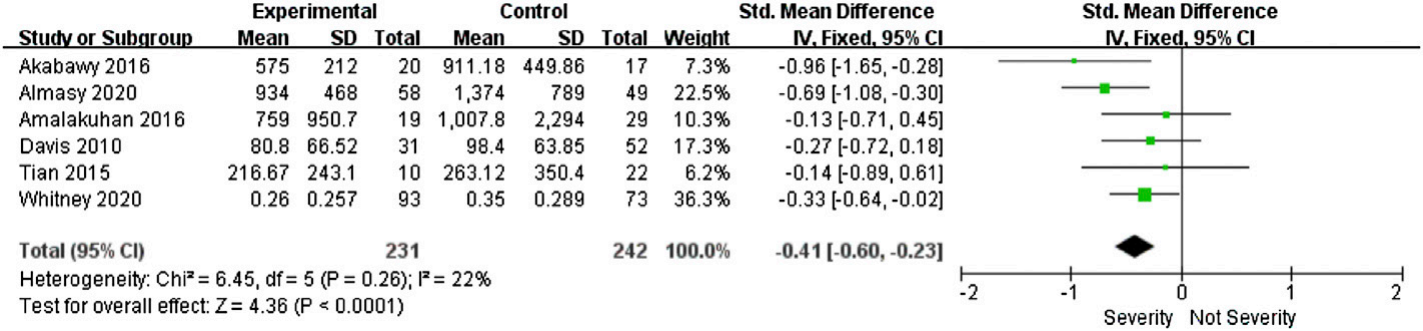
Forest plots of VEGF (sever sepsis-sepsis).

Six studies (Davis et al., 2010; Tian et al., 2015; Akabawy et al., 2016; Amalakuhan et al., 2016; Almasy et al., 2020; Whitney et al., 2020) reported a correlation between VEGF and the severity of sepsis. Due to the good homogeneity between studies (I^2^ = 22%, *p* = 0.26), we used the fixed effect model. Meta-analysis showed that VEGF was significantly higher in severe sepsis than in sepsis (SMD = -0.41, 95%CI-0.6∼-0.23, *p* = 0.0002) (Figure 3). Sensitivity analysis showed that the results remained stable after sequentially removing a single study. It confirmed that high VEGF was associated with more severe sepsis.

### 3.4 VEGF predicts mortality in patients with sepsis

**FIGURE 4:**
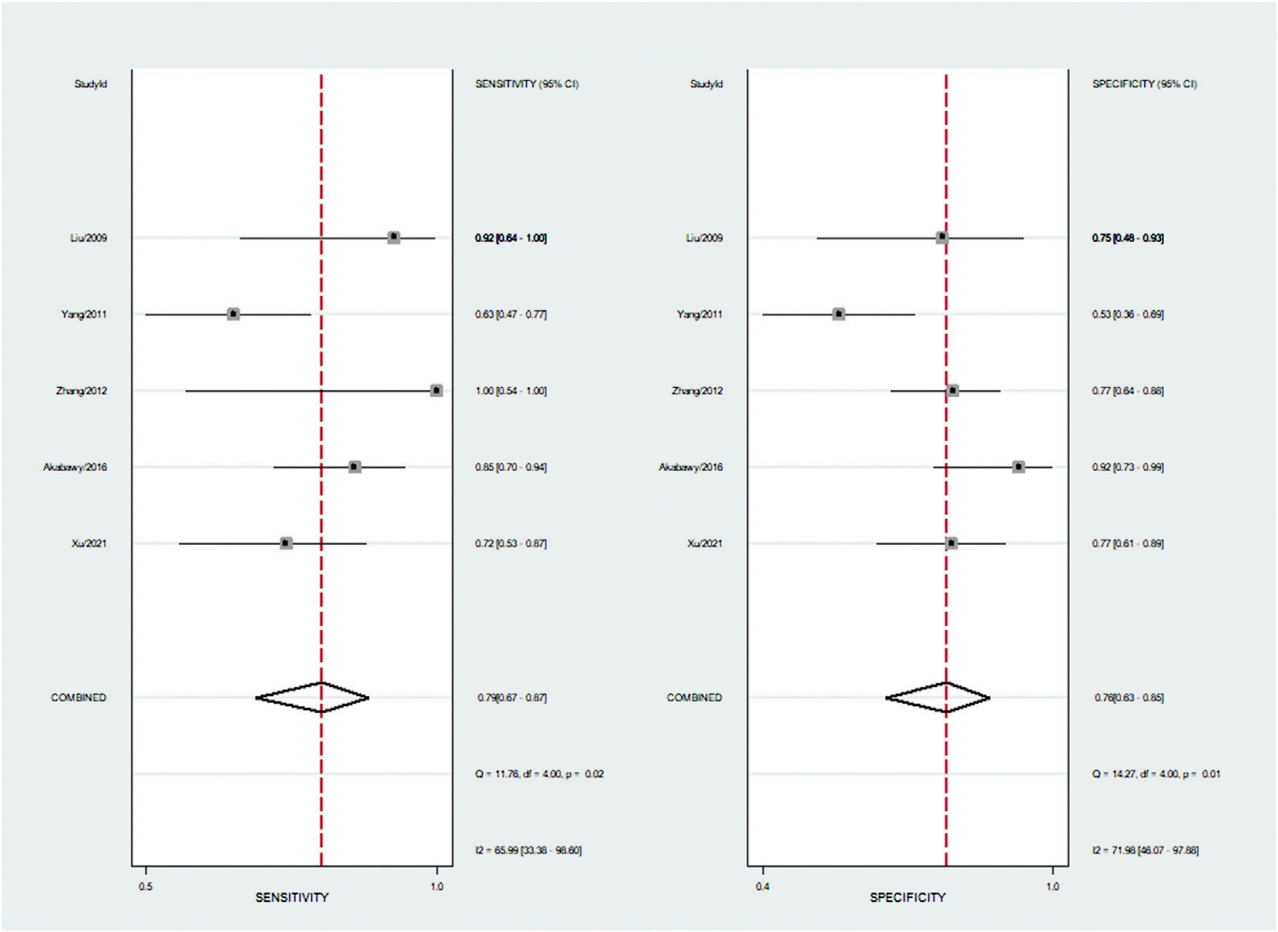
Summary of sensitivity and specificity.

According to the data extracted from five reports (Liu et al., 2009; Yang et al., 2011; Zhang et al., 2012; Akabawy et al., 2016; Xu, 2021), the pooled sensitivity and specificity were 0.79 (95%CI 0.67–0.87) and 0.76 (95%CI 0.63–0.85). Figure 4. The area under the SROC curve is 0.84 (95%CI 0.81–0.87). (Supplementary Figure S3).

## 4 Discussion

Currently, the potential of inflammatory biomarkers to predict adverse outcomes in severe sepsis and septic shock patients remains uncertain (Dellinger et al., 20122013)^,^ (Reinhart et al., 2012). A study (Pregernig et al., 2019) evaluated the prognostic value of six biomarkers in sepsis patients and found that Ang-1, Ang-2, and suPAR had higher predictive value. The role of VEGF in this aspect has not been discussed by systematic review and meta-analysis. VEGF has important pro-angiogenic activity and participates in the regulation of normal and pathological angiogenesis (Melincovici et al., 2018). VEGF can induce leakage of blood vessels, and its increased expression can promote vascular hyperpermeability, edema and tissue damage (Wang et al., 2020). In sepsis, VEGF leads to vascular leakage and enhanced host response (Schuetz et al., 2011). At present, many studies have confirmed that VEGF can predict the prognosis of sepsis, but some studies believe that VEGF is not associated with the prognosis of sepsis (Koskela et al., 2017)^,^ (Karlsson et al., 2008).

Our meta-analysis results suggest that the encouraging prognostic value of VEGF in patients with sepsis. Higher VEGF is associated with higher mortality in sepsis. VEGF in the non-survival group of sepsis was significantly higher than that in the survival group, and in the severe sepsis group was significantly higher than that in the sepsis group. Sensitivity analysis suggested that our results were stable. VEGF has a high predictive accuracy in sepsis mortality, with AUC of 0.84, pooled sensitivity of 79%, and pooled specificity of 76%.

Our study has the following advantages: This study is the first meta-analysis of the prognostic value of VEGF in sepsis. To avoid the impact of other interventions on results, only observational studies were included. Most of the studies were prospective and the quality of the included studies was high. There were a large number of included studies and a large sample size. Sepsis was clearly defined in the included studies. Eligible languages include English and Chinese, which makes the included studies more comprehensive.

This study had some limitations. We detected substantial heterogeneity between studies. Meta-regression found that the sample size of the study might be the source of some heterogeneity, which could not fully explain the source of heterogeneity. Funnel plot indicates that publication bias may exist. We are still unable to explain some studies (Xu et al., 2013) suggesting that VEGF in the non-survival group of sepsis patients is lower than that in the survival group, which is contrary to the results of our meta-analysis.

## Data Availability

The original contributions presented in the study are included in the article/Supplementary Material, further inquiries can be directed to the corresponding author.
