# Supplementary figures and images for: Prognostic role of elevated VEGF in sepsis: A systematic review and meta-analysis
Source: Front Physiol. 2022 Jul 22;13:941257. doi: 10.3389/fphys.2022.941257 (PMC9355294; doi:10.3389/fphys.2022.941257)

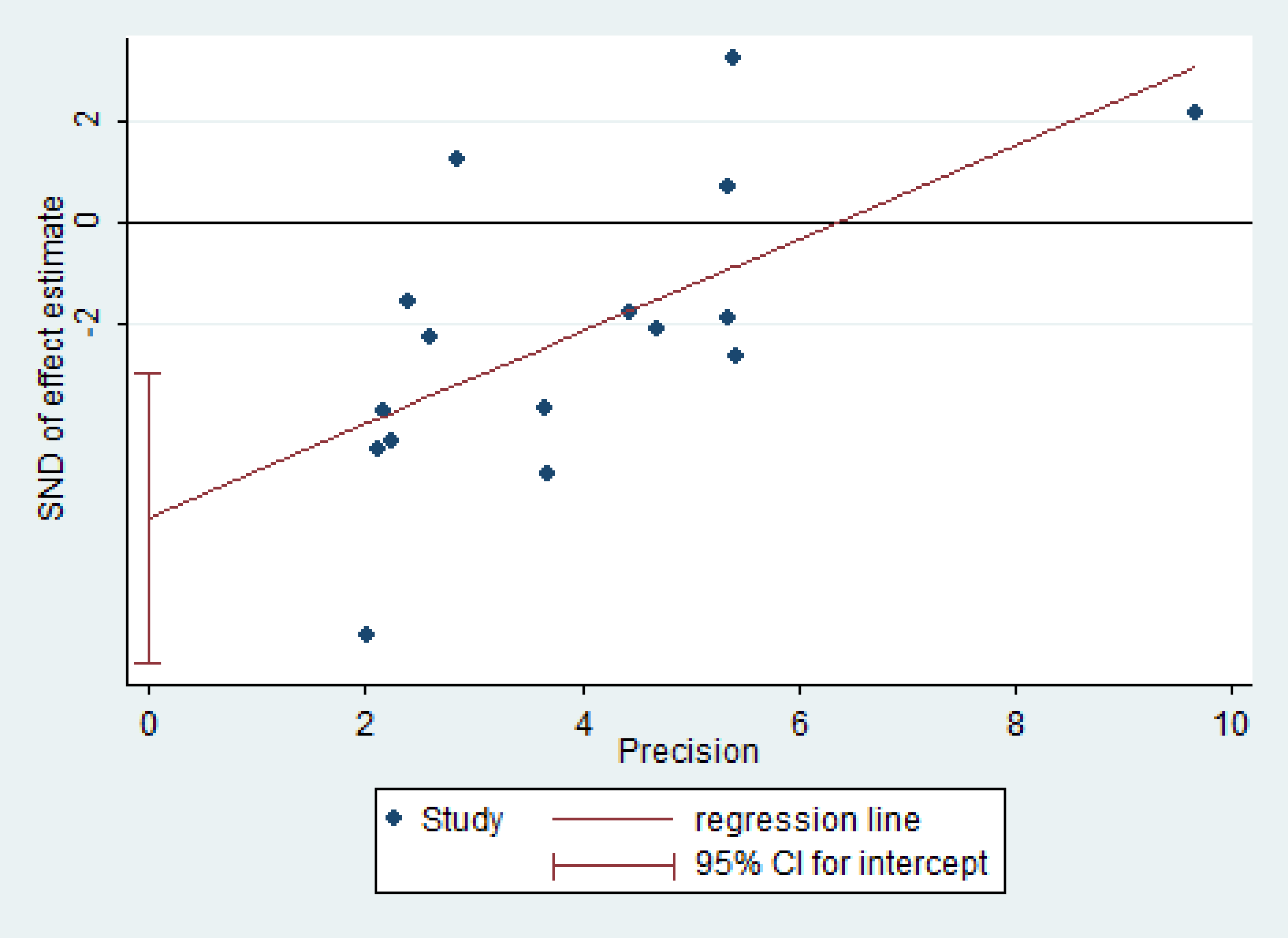

Supplement: Supplementary file 2 [file Image1.TIF]
