# Supplementary material for: Prognostic role of elevated VEGF in sepsis: A systematic review and meta-analysis
Source: Front Physiol. 2022 Jul 22;13:941257. doi: 10.3389/fphys.2022.941257 (PMC9355294; doi:10.3389/fphys.2022.941257)
Supplement: Supplementary file 1 [file DataSheet1.ZIP › Supplementary_Material.docx]

Supplementary Material

# 1 Supplementary Tables

Supplementary Table 1 Search strategy

|  | **Search terms** |
| --- | --- |
|  | ***PubMed*** |
| **#3** | Search #1 AND #2 |
| **#2** | (((((((((((Vascular Endothelial Growth Factor[Title/Abstract]) OR (VEGF[Title/Abstract])) OR (Vascular Endothelial Growth Factor A[Title/Abstract])) OR (Vascular Endothelial Growth Factor A[Title/Abstract])) OR (VEGFA protein, human[Title/Abstract])) OR (Vascular Endothelial Growth Factor Receptor-2[Title/Abstract])) OR (Vascular Endothelial Growth Factor Receptor-1[Title/Abstract])) OR (Receptors, Vascular Endothelial Growth Factor[Title/Abstract])) OR (VEGFB protein, human[Title/Abstract])) OR (Vascular Endothelial Growth Factor D[Title/Abstract])) OR (Vascular Endothelial Growth Factor C[Title/Abstract])) |
| **#1** | (((((sepsis[Title/Abstract]) OR (septic[Title/Abstract])) OR (Septic shock[Title/Abstract])) OR (Severe sepsis[Title/Abstract]))) |
|  | ***Web of Science*** |
| **#3** | Search #1 AND #2 |
| **#2** | TS= (Vascular Endothelial Growth Factor OR VEGF* OR Vascular Endothelial Growth Factor A OR Vascular Endothelial Growth Factor B OR VEGFA protein, human OR Vascular Endothelial Growth Factor Receptor-2 OR Vascular Endothelial Growth Factor Receptor-1 OR Receptors, Vascular Endothelial Growth Factor OR VEGFB protein, human OR Vascular Endothelial Growth Factor D OR Vascular Endothelial Growth Factor C) |
| **#1** | TS= (sepsis OR septic OR Septic shock OR Severe sepsis) |
|  | ***Cochrane Library*** |
| **#3** | #1 and #2 |
| **#2** | Vascular Endothelial Growth Factor*:kw,ti,ab or "VEGF*":kw,ti,ab or "Vascular Endothelial Growth Factor A*":kw,ti,ab or "Vascular Endothelial Growth Factor B*":kw,ti,ab or "VEGFA protein, human*":kw,ti,ab or "Vascular Endothelial Growth Factor Receptor-2*":kw,ti,ab or "Vascular Endothelial Growth Factor Receptor-1*":kw,ti,ab or "Receptors, Vascular Endothelial Growth Factor*":kw,ti,ab or "VEGFB protein, human*":kw,ti,ab or "Vascular Endothelial Growth Factor D*":kw,ti,ab or "Vascular Endothelial Growth Factor C*":kw,ti,ab |
| **#1** | sepsis:kw,ti,ab or septic:kw,ti,ab or Septic shock:kw,ti,ab or Severe sepsis:kw,ti,ab |
|  | ***EMBASE*** |
| **#3** | #1 and #2 |
| **#2** | 'vascular endothelial growth factor':kw,ab,ti OR 'vascular endothelial growth factor a':kw,ab,ti OR vegf:kw,ab,ti OR 'vascular endothelial growth factor b':kw,ab,ti OR 'vegfa protein, human':kw,ab,ti OR 'vascular endothelial growth factor receptor-2':kw,ab,ti OR 'vascular endothelial growth factor receptor-1':kw,ab,ti OR 'receptors, vascular endothelial growth factor':kw,ab,ti OR 'vegfb protein, human':kw,ab,ti OR 'vascular endothelial growth factor d':kw,ab,ti OR 'vascular endothelial growth factor c':kw,ab,ti |
| **#1** | sepsis:ab,ti,kw OR septic:ti,ab,kw OR 'septic shock':ti,ab,kw OR 'severe sepsis':ti,ab,kw |
|  | ***China National Knowledge Infrastructure*** |
| **#3** | #1 and #2 |
| **#2** | TS= (Vascular Endothelial Growth Factor OR VEGF* OR Vascular Endothelial Growth Factor A OR Vascular Endothelial Growth Factor B OR VEGFA protein, human OR Vascular Endothelial Growth Factor Receptor-2 OR Vascular Endothelial Growth Factor Receptor-1 OR Receptors, Vascular Endothelial Growth Factor OR VEGFB protein, human OR Vascular Endothelial Growth Factor D OR Vascular Endothelial Growth Factor C) |
| **#1** | TS= (sepsis OR septic OR Septic shock OR Severe sepsis) |

# 2 Supplementary Figures

**Supplementary Figure 1.** Meta-regression analyses

**Supplementary Figure 2.** Funnel plot

**Supplementary Figure 3.** SROC
